# Early Post-Eclosion Physiological Remodelling in *Tenebrio molitor*: Body Condition, Cuticle Tanning and Immune Maturation During the First Week of Adult Life

**DOI:** 10.3390/insects17080832

**Published:** 2026-08-11

**Authors:** Maria Luigia Vommaro, Anita Giglio

**Affiliations:** 1 Department of Biology, Ecology and Earth Science, University of Calabria, 87036 Rende, Italy; anita.giglio@unical.it; 2 Joint Institute for Individualisation in a Changing Environment (JICE), University of Münster, 48149 Münster, Germany; 3 Joint Institute for Individualisation in a Changing Environment (JICE), Bielefeld University, 33615 Bielefeld, Germany

**Keywords:** body condition, cuticular darkness, haemocyte, immunocompetence, mass-rearing, phenoloxidase, sexual dimorphism, sexual maturity, life-history traits, yellow mealworm

## Abstract

Early adult life represents a critical developmental window during which insects undergo profound physiological reorganization. Despite the economic importance of the yellow mealworm, *Tenebrio molitor* L., this transition remains poorly characterized/understood. This study provides an integrated characterization of the energetic, cuticular and immunological remodelling that occurs during the first week post-emergence, a critical developmental window with important implications for experimental standardization and mass-rearing optimization. Body mass, Scaled Mass Index (SMI), and cuticular tanning trajectories were monitored at 0, 1, 5, and 7 days, along with cellular (tsotal haemocyte count, THC) and humoral (phenoloxidase activity, PO) immunocompetence at 2 and 7 days in both sexes. Newly emerged adults underwent an energetically demanding early phase characterized by significant proportional mass loss, followed by stabilization and improved SMI by day 7. In parallel, THC increased more than twofold, while the humoral defences remained stable. Cuticle melanization followed a synchronized age-dependent trajectory in both sexes, reaching structural stabilization by the end of the first week. Correlation analysis revealed sex-specific patterns of resource allocation. Females showed a positive association between initial body condition and mature PO activity. Conversely, males exhibited a negative trend between cuticle tanning effort and PO activity, suggesting a clear competition for shared physiological resources. Overall, our findings identify early adulthood as a phase of coordinated physiological remodelling, during which energetic reorganization, cuticle maturation, and cellular immune expansion are synchronized to establish adult functional competence.

## Simple Summary

The yellow mealworm (*Tenebrio molitor*) is a key insect used globally as a sustainable, nutritious food. While its larval growth has been extensively studied, we know little about its early development. This study aimed to map how young adult beetles mature during the critical seven-day period after the metamorphosis. Early adult life in *Tenebrio molitor* is a coordinated physiological transition during which energetic reserves are reorganized, cellular immunity is expanded, the cuticle reaches functional maturity, and sex-specific resource allocation strategies begin to emerge. The findings contribute to the advancement of knowledge in the fields of beetle biology and physiology. This knowledge allows insect farmers to improve their breeding protocols, ensuring healthier populations and more efficient production of high-quality protein to help feed the world sustainably.

## 1. Introduction

Early adult life represents a critical developmental transition during which insects complete the establishment of adult physiological functions. Although metamorphosis culminates in adult eclosion, newly emerged individuals are not yet physiologically mature. Instead, they undergo a short but crucial period of structural, metabolic, and immunological reorganization [1] that determines their ability to survive, reproduce, and cope with environmental challenges [2,3,4]. Despite its biological importance, this early post-eclosion maturation window remains poorly characterized in many insect species.

The yellow mealworm, *Tenebrio molitor* L. (Coleoptera: Tenebrionidae), is one of the most widely exploited species in industrial insect farming for sustainable protein production [1,5] and for its ability to bioconvert agro-industrial by-products and degrade plastic waste in the circular economy [6,7]. Its life cycle comprises a 10–12 week larval period with 14 to 18 instars, followed by a 7–8-day pupal stage before adult eclosion [8]. Even though extensive research has been focused on larval growth and nutritional profiles [5,9,10,11], anatomy and metamorphosis [12,13], immunity [14,15,16], and the provision of standardized guidelines for the use of *T. molitor* as a model species in experimental biology [17,18,19], there is currently a significant research gap regarding the physiological events occurring immediately after adult eclosion, despite representing the transition during which newly emerged beetles acquire full adult functionality.

In holometabolous insects, early adult life is characterized by intense physiological activity supported almost exclusively by energetic reserves accumulated during larval development [1]. During this phase, body mass and body condition provide complementary indicators of energetic status, with the latter offering a more reliable estimate of energy reserves because it accounts for structural body size [11,20,21,22]. Monitoring changes in body condition during early adulthood may therefore provide valuable insight into the energetic costs associated with post-eclosion maturation.

Among the most energetically demanding developmental processes occurring after eclosion is cuticle maturation. In *T. molitor* adults, cuticular tanning combines sclerotization (hardening) and melanization (darkening) processes, which progressively transform the newly emerged, soft exoskeleton into a rigid protective barrier [23]. This process also requires a substantial metabolic investment, relying heavily on tyrosine-derived biochemical pathways that are linked to immune function and pathogen resistance [14,24]. Besides providing structural support, the insect cuticle is a multifunctional barrier providing protection against desiccation, mechanical injury, and pathogens [14,25]. As a result, exoskeleton maturation may compete with other physiological functions for limited energetic resources during early adulthood.

In addition, the ability of insects to withstand infection is crucial for their survival. Following cuticle breach, immunocompetence constitutes a critical second line of defence that provides systemic protection [14]. The internal system is constituted by an integrated network of cellular and humoral effectors that function to rapidly identify and neutralize pathogens [14,17,26]. The cellular response is mediated by specialized circulating cells called haemocytes, primarily granulocytes, plasmatocytes, and oenocytoids in *T. molitor* [27], which engage in phagocytosis, nodulation, and the encapsulation of invaders [28]. Humoral immunity involves soluble factors in the haemolymph, most notably the phenoloxidase (PO) cascade, which is responsible for melanization and the production of toxic reactive intermediates at wound sites or around pathogens [29]. This is complemented by the synthesis of antimicrobial peptides (AMPs), such as Tenecins, Attacin, Cecropin, Defensin and Coleoptericin, which are primarily produced by the fat body and provide sustained humoral and other chemical defences [30,31,32]. These energy-demanding traits are tightly coordinated through the neuroendocrine system [33,34] and the allocation of limiting resources, reflecting complex life-history trade-offs between structural armour, internal defensive bolstering, and reproductive readiness [24,26,35]. It has been demonstrated that the primary factor influencing survival under conditions of food limitation in *T. molitor* is initial body mass [36]. This indicates that the sexual maturity transition is a crucial factor in determining the state of mature adults.

Sex-specific life-history strategies are expected to further influence these physiological transitions. Males and females employ distinct resource allocation strategies to optimize their respective levels of fitness, resulting in distinct investment patterns in reproduction and self-maintenance [16,37]. Females invest in traits that favour longevity and sustained reproductive output, often reaching peak fertility during their third week of adulthood [36,38]. Conversely, males reach reproductive competence within the first week after eclosion and may prioritize reproductive effort or sexual attractiveness over somatic maintenance or future immune competence [39,40,41]. These divergent reproductive strategies suggest that the coordination of energetic allocation, cuticle maturation, and immune development may differ between the sexes during early adulthood.

Despite the economic importance of *T. molitor*, the physiological integration of these processes during the early post-eclosion period remains poorly understood. In particular, a comprehensive understanding of how *T. molitor* balances the competing metabolic demands of exoskeleton hardening and the establishment of a robust immune defence and sexual maturation during early adulthood is lacking.

Here, we tested the hypothesis that the first week following adult eclosion represents a coordinated phase of physiological remodelling during which energetic reserves, cuticle maturation, and immune competence are synchronously reorganized. To address this question, we quantified changes in body mass and body condition (Scaled Mass Index), cuticle tanning, cellular (total haemocyte count), and humoral (phenoloxidase activity) immunity throughout the first week of adult life in both females and males. By integrating these complementary physiological traits across the first 7 days post-emergence, this study establishes a comprehensive baseline for early adult maturation in *T. molitor*, providing valuable insights for insect mass-rearing practices, experimental standardization, and future studies of experimental biology using this beetle as a model to investigate the physiological mechanisms underlying adult performance.

## 2. Materials and Methods

### 2.1. Insect Breeding and Rearing Conditions

Specimens of *T. molitor* utilized in this study were obtained from a continuous stock population maintained at the Morpho-functional Entomology Laboratory (University of Calabria, Italy). The stock was reared in controlled environmental conditions, characterized by a relative humidity of 60% to 50%, a natural photoperiod, and a temperature of 25 ± 3 °C. Larvae were reared in open plastic boxes containing organic white wheat flour and bran, which was supplemented periodically with organic fresh fruits and vegetables as a water source. In order to achieve precise synchrony in the process of adult eclosion, pupae were collected on a daily basis from the stock culture. The sex of the individuals was determined by examining the caudal genital papillae under a stereomicroscope (Leica Zoom 2000, Wetzlar, Germany). Thereafter, the pupae were housed separately in distinct boxes to complete their development until metamorphosis.

### 2.2. Experimental Design and Sampling Timeline

Newly emerged adults (post-eclosion, designated as day 0) were isolated and placed individually into 30 mL plastic cups with perforated lids to ensure adequate air exchange. In order to ensure the maintenance of a constant internal moisture level and thereby prevent dehydration, each cup was equipped with a piece of filter paper measuring 2 cm × 2 cm, soaked in sterile phosphate-buffered saline (PBS, 10 mM; Sigma-Aldrich, Milan, Italy), to maintain osmotic balance. The filter paper was replaced at two-day intervals throughout the experiment in order to prevent contamination from mould. The beetles were provided with a standard diet of organic flour (0.05 ± 0.02 g per cup; Belbake, Neckarsulm, Germany) which was replenished on an ad libitum basis. All physiological and immunological samplings were carried out on these isolated individuals at precise, predefined ontogenetic intervals, starting from eclosion (0 d) up to the achievement of full biological maturity at day 7 (7 d).

### 2.3. Body Mass Dynamics and Scaled Mass Index (SMI)

To assess energetic profiles and body condition trajectories, individual body mass was recorded using an analytical balance (Ohaus, Parsippany, NJ, USA. sensitivity ± 0.1 mg) at three chronological stages in both males and females (*n* ≥ 60 per sex): immediately after eclosion (0 d), at day 5 (5 d), and at day 7 (7 d). Proportional relative mass loss was calculated for two consecutive time intervals: Δmass_0–5_; Δmass_5–7_ (*n* = 31 per sex; total observations = 62). Body condition was quantified using the Scaled Mass Index (SMI) computed according to Peig et al. [42], standardizing body mass relative to a linear body size indicator to account for morphometric scaling differences between sexes. The length of the right elytron was measured to estimate body size. Each elytron was measured digitally using ImageJ (version 1.54t, National Institutes of Health, Bethesda, MD, USA) twice, and the average was used for analysis. SMI was calculated using the following formula:

SMI = M_i_ [T_0_/T_i_]^bSMA^

where M_i_ is the body mass of individual (i), T_i_ is the structural dimension (elytron length), T_0_ is the population mean, and bSMA is the slope of the regression between the logarithms of body mass and body size. SMI was calculated for each individual at five-day intervals. The population mean elytron length was used as a constant in SMI calculations across all time points and for both sexes (*n* = 20 females, *n* = 19 males).

### 2.4. Cuticle Tanning Quantification

The progression of cuticular melanization and sclerotization was monitored non-invasively by measuring cuticular luminance. Dorsal images of individual beetles were captured under standardized lighting conditions under a stereomicroscope (Zeiss Stemi SV11, Carl Zeiss Microscopy GmbH, Jena, Germany) and photographed using a digital camera (Optica C-P8) at precise ontogenetic intervals, day 0 (0 d), day 1 (1 d), day 5 (5 d), and day 7 (7 d), for males and females (*n* ≥ 20). The images were processed using digital image analysis software (ImageJ), and the luminance values were extracted from standardized regions of the elytra twice. The degree of cuticular darkness was scored as the luminance intensity on a grey scale ranging from 0 to 255, where 0 represents the darkest shade (maximum melanization), and 255 represents the brightest shade (absence of melanization) [43]. Sample sizes varied by developmental stage depending on randomized block scheduling (days 0 and 7: *n* = 30 per sex; days 1 and 5: *n* = 20 per sex).

### 2.5. Haemolymph Collection, Total Haemocyte Count and Phenoloxidase Activity Assays

To evaluate cellular immune status, haemolymph was extracted from beetles at day 2 and day 7 post-eclosion. Each specimen was punctured at the pro-mesothorax junction utilizing a sterile 29-gauge needle as in Vommaro et al. [44]. A fixed volume of 3 µL of haemolymph was immediately collected via a micropipette and diluted 1:1 (*v*/*v*) in a sterile anticoagulation solution (PBS, 10 mM; Sigma-Aldrich, Milan, Italy, supplemented with 17 mM Ethylenediaminetetraacetic acid disodium salt dihydrate, EDTA, Sigma-Aldrich) at 4 °C to prevent clotting and melanization. The total haemocyte count (THC) was subsequently quantified using a Bürker haemocytometer chamber (Carlo Erba, Milan, Italy) under a Zeiss Primo Star light microscope (Carl Zeiss Microscopy GmbH, Jena, Germany). Final cellular concentrations were expressed as the number of circulating cells per millilitre of haemolymph at 2 and 7 days in both males and females. For cellular immunity, THC was successfully quantified for 12 males and 12 females at day 2 and 10 males and 8 females at day 7 (*n* = 42 total observations).

Humoral immunocompetence was evaluated by measuring both basal and total phenoloxidase (PO) activity at day 2 and day 7 post-eclosion in males and females (*n* = 15 biological replicates per sex per time point; total observations = 60; with two technical replicates per sample). Haemolymph samples (4 µL) were collected as mentioned before and diluted 1:15 in sterile phosphate buffer (PBS, 10 mM; Sigma-Aldrich, Milan, Italy) at 4 °C and centrifuged at 10^4^ rpm for 5 min. The supernatant (cell-free haemolymph–PBS mixture) was collected and stored at −20 °C until biochemical analysis. PO activity was measured spectrophotometrically by monitoring the conversion of L-dihydroxyphenylalanine (L-DOPA) into dopachrome at 492 nm and 25 °C for 60 min at 15 s intervals using a Thermo Scientific Multiskan FC plate reader (Thermo Fisher Scientific, Waltham, MA, USA), as in Naccarato et al. [43]. Enzymatic assays were performed in duplicate using sterilized 96-well microtiter plates. The final values represent the mean of two technical replicates. To determine total PO activity via pro-PO zymogen activation, 10 µL of the cell-free extract was mixed with 10 µL of LPS (lipopolysaccharides from *Escherichia coli* O127:B8; Sigma-Aldrich, Milan, Italy) dissolved in cold, sterile PBS (1 mg/mL) and incubated at room temperature for 5 min. For basal PO activity, the LPS activator was replaced by mixing 10 µL of the cell-free extract with 10 µL of sterile PBS. The enzymatic reaction was initiated by adding 80 µL of L-DOPA (3 mg/mL in PBS; (3-(3,4-dihydroxyphenyl)-L-alanine; L-3-hydroxytyrosine, Sigma-Aldrich) to each well. Negative control (blank) wells contained identical volumes of PBS instead of the haemolymph mixture. Enzyme activity was calculated from the linear slope of the reaction curve during its maximum velocity phase (Vmax value), evaluated 15 min after the reaction onset by deliberately excluding the initial lag and final plateau phases, and expressed as the change in absorbance per minute (ΔA_492_/min).

### 2.6. Statistical Analyses

Statistical analyses and data visualization were performed using R software (version 4.5.0) [45], with the tidyverse, lme4, lmerTest, and ggplot2 packages. Normality and homoscedasticity were verified via Shapiro–Wilk and Levene’s tests, respectively. To account for repeated measures across adult ontogeny, absolute body mass, Scaled Mass Index (SMI), and cuticle tanning luminance were analyzed using Linear Mixed-Effects Models (LMMs) with individual ID as a random intercept and Satterthwaite-approximated degrees of freedom. Fixed factors included sex (male, female) and time (days 0, 5, 7 for mass/SMI; days 0, 1, 5, 7 for luminance), plus their interaction. Paired relative mass loss (Δmass_0–5_ vs Δmass_5–7_) was evaluated using a non-parametric Wilcoxon signed-rank test to accommodate extreme physiological outliers, which were retained in all models to preserve data integrity.

For independent destructive sampling, THCs were evaluated using Two-Way ANOVA (sex × time). Humoral immunocompetence was assessed via Three-Way ANOVA including sex, time, and enzyme status (basal vs. total PO) with all two- and three-way interactions. Post hoc pairwise comparisons for all linear models and ANOVAs were conducted using Tukey’s HSD test via emmeans.

Pairwise physiological coordination across adult ontogeny was evaluated via Pearson correlation coefficients with two-tailed t-tests. These were restricted to a multi-trait subset (*n* ≥ 17) where body mass, SMI, cuticle luminance, and PO activity were simultaneously recorded, with tanning and energetic coordinates standardized over the day 0 to 7 interval. Significance was set at *p* < 0.05 across all tests.

## 3. Results

### 3.1. Body Mass and Scaled Mass Index (SMI) Dynamics

**Figure 1:**
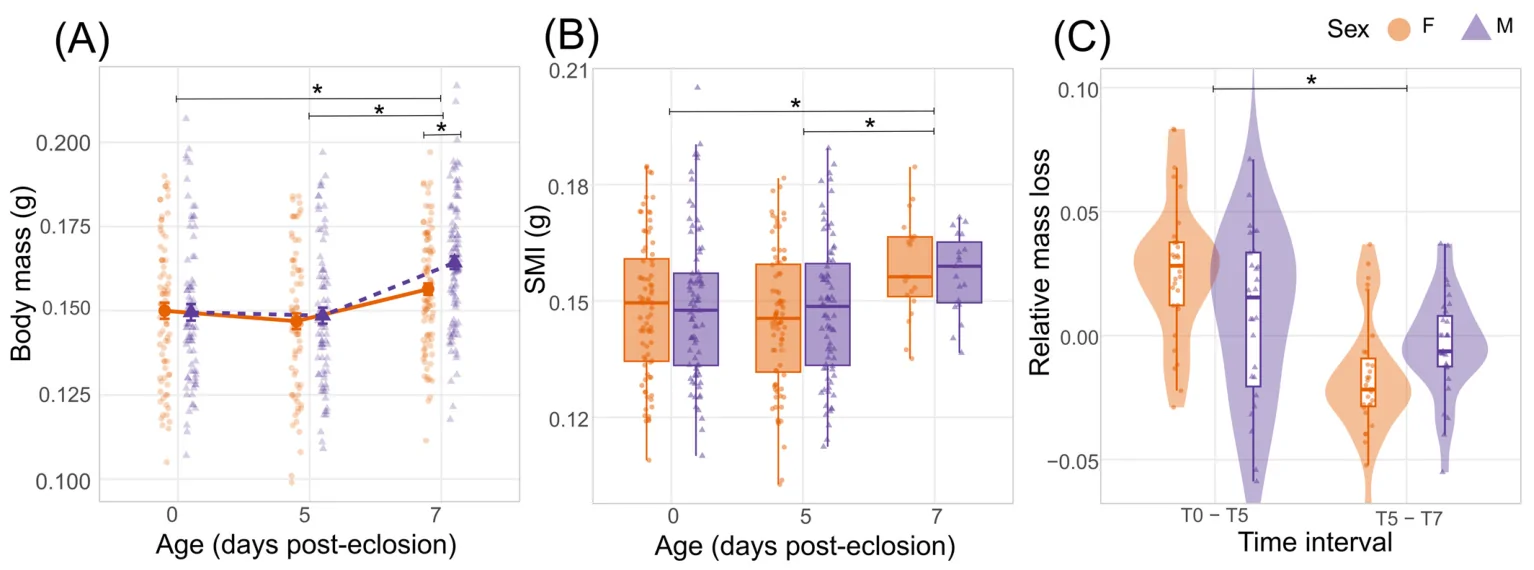
Physiological variation in energetic body mass allocation and metabolic parameters in *Tenebrio molitor* during early adult maturation. Total body mass fluctuations (**A**) and progression of the Scaled Mass Index (**B**) recorded at 0, 5, and 7 days post-eclosion across female and male cohorts. (**C**) Relative mass loss profiles evaluated between the specific maturation intervals Δmass_0–5_; Δmass_5–7_), highlighting the primary energetic investment phase occurring immediately post-eclosion. Asterisks indicate statistically significant differences (*p* < 0.05).

The LMM analysis on absolute body mass (Figure 1A) revealed a highly significant main effect of time (*F*_2, 501.11_ = 6.31, *p* = 0.002), and sex (*F*_1, 507.45_ = 6.14, *p* = 0.014). Post hoc comparisons showed that sexual dimorphism emerged dynamically: absolute biomass was uniform at eclosion (*p* = 0.714) and during the intermediate stage (T5, *p* = 0.250) but diverged significantly by day 7, with males becoming heavier than females (T7, *p* = 0.003; Figure 1A). The non-significant sex × time interaction (*F*_2, 415.68_ = 1.97, *p* = 0.141) confirmed that both cohorts shared a synchronized trajectory, with mass staying stable from day 0 to 5 (*p* = 0.507) but increasing significantly from day 0 to 7 (*p* = 0.027) before peaking sharply between days 5 and 7 (*p* = 0.001).

When mass was standardized into relative body condition via the SMI (Figure 1B), the LMM confirmed a significant variation over time (*F*_2, 318.16_ = 5.23, *p* = 0.006). Relative condition remained stationary early on (0 d vs. 5 d: *p* = 0.528) but improved robustly at day 7 compared to both baseline (0 d vs. 7 d: *p* = 0.029) and the intermediate stage (d5 vs. 7 d: *p* = 0.004). Notably, the specific model predictors indicated that the transition to day 7 exerted a significant positive effect on individual body condition relative to the baseline (*t* = 2.15, *p* = 0.033; Figure 1B). Conversely, the main effect of sex was entirely non-significant (*F*_1, 309.55_ = 0.015, *p* = 0.902), and no significant interaction between sex and time was detected (*F*_2, 308.80_ = 0.33, *p* = 0.722). This statistical shift demonstrates that the dimorphism observed in absolute mass is purely allometric (driven by differences in structural size differences), whereas relative body condition and energetic scaling remain uniform between the sexes throughout maturation (Figure 1B).

Regarding proportional weight fluctuations (Figure 1C), a Wilcoxon signed-rank test confirmed a significant shift between intervals (*V* = 1558, *p* < 0.001). The population experienced generalized, positive proportional mass loss during the initial post-eclosion phase (Δmass_0–5_), which then contracted and stabilized around zero during the subsequent interval (Δmass_5–7_), marking the cessation of active mass depletion (Figure 1C).

### 3.2. Cellular Immune Density

**Figure 2:**
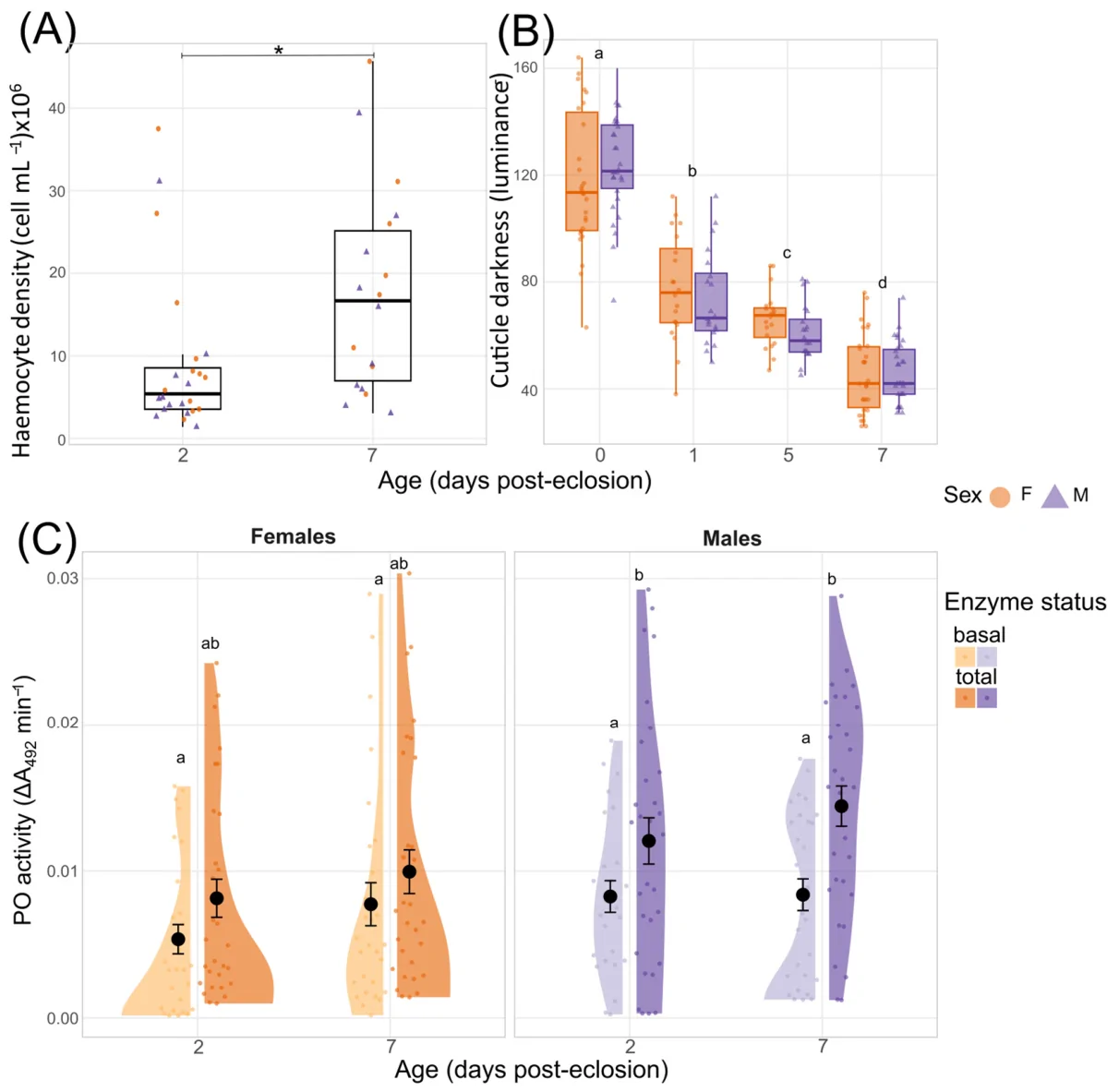
Ontogenetic trajectories of cellular immunity, enzymatic cascades, and cuticular melanization in ageing *Tenebrio molitor* adults. (**A**) Total Haemocyte Count (THC, cells/mL) from 2 to 7 days post-eclosion. (**B**) Cuticle tanning progression quantified via structural reflectance (luminance units) at four successive maturation intervals (newly eclosed 0, 1, 5 and 7 days post-eclosion). (**C**) Phenoloxidase (PO) activity kinetics measured as the maximum reaction velocity (ΔA_492_/min) comparing basal activated pro-enzymes (lighter shade) with fully activated total (darker shade) PO activity across distinct maturation timepoints (2 and 7 days post-eclosion) in both females and males. Different lowercase letters and asterisks indicate statistically significant differences (*p* < 0.05).

The Two-Way ANOVA conducted on the THCs demonstrated a highly significant main effect of age (F_1, 38_ = 16.29, *p* < 0.001). A sharp, more than two-fold increase in the concentration of circulating immune cells was observed as beetles transitioned from young immature (2 days) to mature adults (7 days post-eclosion), with an overall mean increase of 11.00 × 10^6^ cells/mL (*p* < 0.001; Figure 2A). Conversely, the main effect of sex (*F*_1, 38_ = 1.17, *p* = 0.287) and the interaction between sex and time (F_1, 38_ = 0.39, *p* = 0.536) were strictly non-significant; this confirms that the systemic up-regulation of circulating haemocytes represents a generalized, non-dimorphic physiological feature shared equally by both sexes during early adulthood (Figure 2A).

### 3.3. Cuticle Darkness and Phenoloxidase Activity

The progression of exoskeleton maturation was first assessed by measuring changes in tegumental lightness (Luminance) across early adult life (Figure 2B). The LMM on cuticle pigmentation revealed a dominant main effect of time (*F*_3, 170.99_ = 244.46, *p* < 2.2 × 10^−16^), while the effect of sex (*F*_1, 168.64_ = 0.04, *p* = 0.847) and the sex*time interaction (*F*_3, 162.70_ = 1.16, *p* = 0.328) were entirely non-significant. This demonstrates that exoskeleton melanization kinetics operate uniformly between the sexes. Tukey’s post hoc comparisons confirmed a highly synchronized, two-step darkening cascade for both cohorts: an acute, rapid decline in luminance occurred within the first 24 h post-eclosion (0 d vs. 1 d; females: *p* < 0.001; males: *p* < 0.001), followed by a stationary phase until day 5 (1 d vs. 5 d; *p* > 0.05 for both sexes) and a secondary significant darkening phase by day 7 (5 d vs. 7 d; females: *p* = 0.0003; males: *p* = 0.024; Figure 2B).

For humoral immunocompetence, the Three-Way ANOVA revealed no significant two-way or three-way interactions among variables (*p* > 0.195 for all interaction terms), pointing to independent physiological drivers. Highly significant main effects emerged for enzyme status (*F*_1, 221_ = 15.59, *p* = 0.0001), with total PO levels sharply exceeding basal levels and sex (*F*_1, 221_= 10.86, *p* = 0.001), while time was non-significant (*F*_1, 221_ = 3.05, *p* = 0.082). Slice-wide comparisons clarified that while basal PO remained equivalent between sexes at all time points (2 d basal: *p* = 0.138; 7 d basal: *p* = 0.733), a robust sexual dimorphism favoured males in total PO capacity at both day 2 (2 d total: *p* = 0.034) and day 7 (7 d total: *p* = 0.016). In contrast, temporal trends remained entirely stationary for both sexes and activation states between days 2 and 7 (*p* > 0.05).

### 3.4. Sex-Specific Correlative Trade-Offs in Resource Allocation

**Figure 3:**
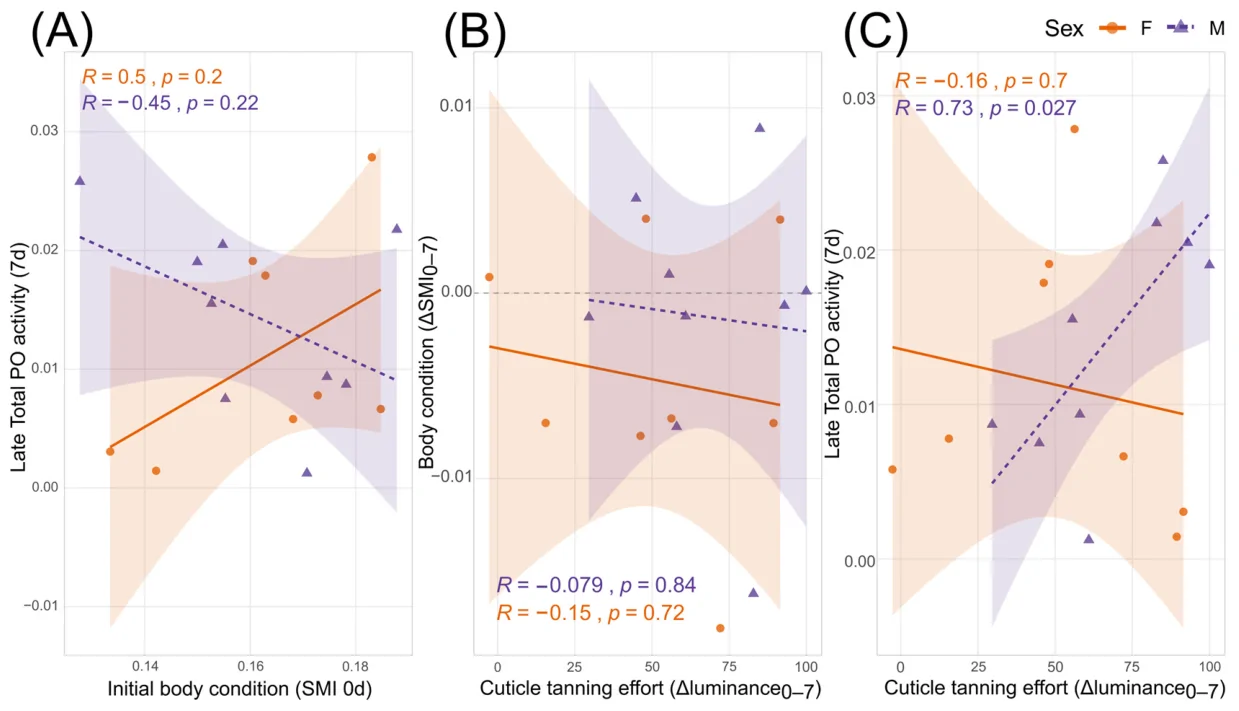
Sex-specific correlation analyses evaluating physiological and immunological maturation pathways in *Tenebrio molitor*. (**A**) Correlation between the initial structural condition (SMI 0 d) and the mature humoral immunity peak at day 7 (Total PO activity 7 d). (**B**) Correlation between total cuticular tanning effort (ΔLuminance_0–7_) and the absolute change in relative body condition (ΔSMI_0–7_). (**C**) Correlation between cuticular tanning effort (ΔLuminance_0–7_) against final potential immune investment in PO activity (totalPO 7 d). Individual data points are stratified by sex (females: orange circles; males: purple triangles), with lines representing linear regression trends and shaded areas indicating 95% confidence intervals.

**Figure A1:**
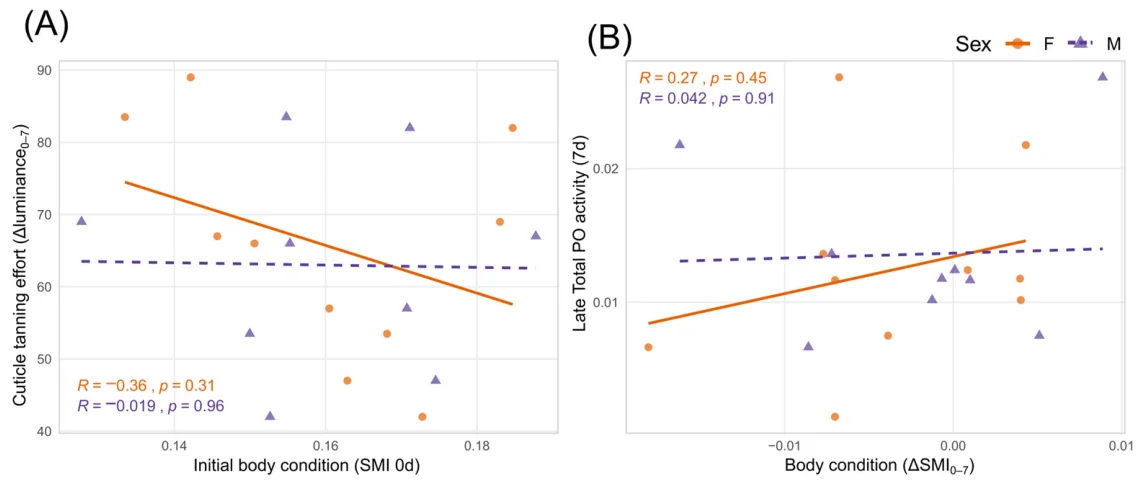
Pearson correlation analyses evaluating potential physiological and immunological trade-offs in *Tenebrio molitor* during early adult maturation, stratified by sex (females and males). (**A**) Correlation between the initial structural condition (SMI 0 d) and the cuticular tanning effort (ΔLuminance_0–7_). (**B**) Correlation between PO activity at 7 d post-eclosion and the absolute change in relative body condition (ΔSMI_0–7_).

Pearson correlation analyses revealed that initial structural condition (SMI_0d_) did not significantly correlate with total tanning effort (ΔLuminance_0–7_; Females: *R* = −0.36, *p* = 0.31; Males: *R* = −0.019, *p* = 0.96; Figure A1A) or peak mature immunity (total PO activity_7d_). However, the latter relationship showed a non-significant descriptive divergence: females displayed a positive trend (*R* = 0.50, *p* = 0.20; Figure 3A), while males showed a moderate negative trend (*R* = −0.45, *p* = 0.22; Figure 3A).

Regarding the somatic cost associated with exoskeleton darkening (Figure 3B), total tanning effort (ΔLuminance_0–7_) did not correlate with changes in body condition (ΔSMI_0–7_) for either sex (Females: *R* = −0.15, *p* = 0.72; Males: *R* = −0.079, *p* = 0.84; Figure 3B). Consistently, final immune potency (total PO activity_7d_) showed no correlation with net condition changes (ΔSMI_0–7_) across either cohort (Females: *R* = 0.27, *p* = 0.45; Males: *R* = 0.042, *p* = 0.91; Figure A1B), confirming a lack of visible trade-offs between maturation and somatic growth.

Crucially, a stark sexual dimorphism emerged regarding direct pathway substrate competition (ΔLuminance_0–7_ vs. total PO activity_7d_; Figure 3C). While females exhibited a weak, entirely non-significant relationship (*R* = −0.16, *p* = 0.70), males displayed a strong, significant negative correlation (*R* = −0.73, *p* = 0.027).

## 4. Discussion

Our findings demonstrate that the first week following adult eclosion represents a coordinated phase of physiological remodelling rather than a simple continuation of metamorphosis. During the post-eclosion maturation, *T. molitor* adults undergo a tightly synchronized transition in which energetic reserves, structural maturation, and immune competence are progressively reorganized until a stable physiological state is reached by approximately seven days after emergence. Rather than developing independently, these processes appear to be integrated components of a common developmental programme that prepares newly emerged beetles for adult life in a window of 7 days. The dynamics observed throughout early adulthood strongly support this interpretation. However, early adulthood experienced a generalized proportional loss of body mass across both sexes during the first five days after eclosion. Body condition, estimated using the Scaled Mass Index (SMI), improved significantly by day 7. This indicates that the early reduction in biomass does not reflect a deterioration of physiological status but rather the strategic utilization of larval energetic reserves to complete adult maturation. The absence of sexual differences in body condition indicates that males and females maintain comparable energetic status during early adulthood despite differences in absolute size. Thus, the observed dimorphism is primarily allometric rather than physiological, reflecting differences in body size rather than unequal energetic allocation, as observed in the yellow dung fly *Scathophaga stercoraria*, where sex-specific energy reserve differences also vanish when standardizing for structural size [22]. These findings indicate that individuals reached peak structural quality upon maturity, potentially supported by the extensive internal tissue remodelling that occurs during the pupal stage to prepare the adult for its reproductive role [13]. Previous studies showed in *T. molitor* that larger beetles generate significantly heavier individual larvae, and selecting for larger size, particularly in fathers, can enhance overall farm productivity [46]. Future studies quantifying specific energy reserves (such as total lipids, carbohydrates, and glycogen storage) will provide deeper insights into metabolic costs during this phase.

The marked reduction in body mass coincided with the period of maximal cuticular tanning, emphasizing the substantial energetic investment required to transform the newly emerged exoskeleton into a mechanically resistant and physiologically functional structure. Cuticular sclerotization and melanization demand substantial amino acids and metabolic energy [47], driving this immediate mass loss followed by progressive stabilization until day 7. This synchronized endpoint in cuticle tanning suggests that reaching a highly melanized cuticle represents a strict evolutionary constraint shared equally by both sexes [48]. The adaptive significance of this investment extends well beyond cuticle pigmentation itself. A fully melanized exoskeleton is essential for multiple biological functions, including (i) resistance to desiccation, as the cuticle serves as a multifunctional device to protect insects from dehydration [25]; (ii) mechanical protection, as it provides a robust physical barrier that acts as a first line of defence against wounding and external stressors [49]; and (iii) and defence against pathogens, as the degree of cuticular melanization in *T. molitor* is a strong indicator of resistance to entomopathogenic fungi, with darker beetles possessing thicker, less porous cuticles that are more resistant to microbial penetration [50]. A fully tanned exoskeleton is also a fixed physiological priority for long-term survival [49]. The timing of this maturation also coincides with the peak expression of *TmTH* and *TmDDC*, key genes regulating the synthesis of melanin precursors required for cuticular hardening [23]. Furthermore, while cuticular darkness is a strong indicator of pathogen resistance in *T. molitor*, the degree of melanization ultimately reflects the underlying architecture and thickness of the exocuticle [24]. Crucially, the finding that cuticular tanning effort did not impose a detectable somatic drain on body condition for either sex points to highly efficient internal physiological buffering. Although cuticular melanization and sclerotization are energetically and biochemically expensive [51], *T. molitor* successfully fulfils these requirements without compromising relative body condition during this early ontogenetic window. This capacity to isolate the somatic costs of exoskeleton consolidation from overall condition highlights the species’ metabolic resilience and the strategic importance of its initial energetic reserves [52].

In parallel with exoskeleton maturation, the immune system underwent substantial reorganization, as highlighted by more than a two-fold increase in THC between days 2 and 7. This age-dependent expansion occurred similarly in both sexes, indicating that the establishment of cellular immune competence represents a conserved component of early adult maturation rather than a sex-specific trait. As granulocytes and plasmatocytes mediate key cellular responses, including phagocytosis and encapsulation [14,27], the increase in circulating haemocytes suggests that, as the exoskeleton hardens, the insect scales up immune defences for adult reproductive challenges. During metamorphosis, haemocytes are heavily sequestered for internal tissue remodelling and larval fat body degradation [53]; this could explain their low numbers in early adulthood. As internal organs stabilize and the insect approaches sexual maturity, these cells are recruited into the hemocoel [54], marking a programmed developmental transition into full cellular readiness. The expansion of THC in *T. molitor* mirrors the post-emergence maturation pattern of the invasive ladybird *Harmonia axyridis*, where haemocyte density increases six-fold during the first 8 days of adult life [55]. This “bolstered” adult state contrasts with the developmental trajectories of other orders, such as the cockroach *Blattella germanica*, which exhibits a continuous linear increase in THC throughout its life cycle [56]. This maturation process is likely coordinated by developmental hormones such as 20-hydroxyecdysone (20E), which acts as a dual regulator driving haemocyte differentiation while simultaneously controlling the expression of *TmTH* and *TmDDC* for cuticular tanning [23,34]. This shared endocrine upstream control explains why these distinct processes are so tightly synchronized. Future investigations should incorporate functional immune challenges (e.g., LPS injection or bacterial exposure) to verify whether this day-7 peak state directly translates into enhanced survival or pathogen resistance.

In contrast to the rapid cellular expansion, the humoral immune compartment remains remarkably stable throughout early adult development. The maintenance of a constant PO zymogen reserve suggests that this defence is already fully functional at adult emergence. The PO cascade is a fundamental component of the constitutive, activatable immune response [29], ensuring rapid activation of melanization responses while the soft, newly emerged cuticle remains vulnerable to injury and infection [49]. The stability of PO activity in *T. molitor* during the first week after emergence is distinct from that in other beetles. The ground beetle *Carabus lefebvrei* exhibits high PO activity in tanned larvae and adults but very low levels in the pupal stage [57]. In the Lepidopteran *Spodoptera littoralis*, PO activity is dynamic during maturation, reaching a maximum at pharate stages before dropping after ecdysis [58]. Other beetles, like the burying beetle *Nicrophorus vespilloides*, have more dramatic changes in their immune traits [53]. However, our analysis reveals a substrate-level trade-off exclusive to males. While tyrosine is required for both exoskeleton hardening and internal immunity [24], males allocating greater resources towards extensive cuticular melanization suffer a significant depletion in their final potential immune defence. This sex-specific bottleneck demonstrates that early maturation in male *T. molitor* is strictly constrained by a metabolic trade-off between exoskeleton consolidation and humoral competency. This pattern was not detected in females, which suggests that the distribution of resources during the early stages of maturation differs between the sexes. Our observations display early adulthood as a period of meticulously orchestrated up-regulation, wherein both external armour and internal defences undergo synchronized fortification. The physiological changes observed in *T. molitor* during the first week after emergence are important for the development of many insects. This is also seen in the fruit fly *Drosophila montana*, where life-history traits are “reset” and adjusted after this period to establish the baseline for future fitness [2].

In evaluating the cross-talk between initial energetic reserves and mature humoral capacity, we observed descriptive, non-significant trends suggesting divergent trajectories between the sexes. Although these statistical values reflect an underpowered subset preventing definitive conclusions, they suggest potentially divergent allocation strategies. Females tended to show a positive association between initial reserves and subsequent PO capacity, whereas males hint at a potential constraint. This tentative divergence warrants targeted, high-power validation in future resource-allocation designs. Such sex-specific trajectories are biologically plausible, as female insects frequently face trade-offs between immune investment and egg production during vitellogenesis [26], whereas male reproductive success may depend more strongly on early reproductive activity [16]. Previous studies suggest *T. molitor* males reach reproductive potential as early as two weeks after emerging, whereas females require a more gradual maturation process [59]. Furthermore, Krams et al. [60,61] suggest that males may adopt a terminal investment strategy, maintaining high reproductive effort or sexual attractiveness at the expense of future immune competence. This supports our interpretation that males prioritize rapid exoskeleton stabilization and immediate mating effort, even if immune defences are depleted. Although beetles were reared on a standardized diet, the pronounced physiological plasticity of *T. molitor* [62] suggests that dietary manipulation [1,14,24], particularly supplementation with limiting precursors, such as tyrosine, could substantially alter these developmental trajectories. Moreover, because survival under nutritional stress is primarily determined by absolute body mass rather than immune expenditure [36], the absence of a relationship between tanning effort and body condition further supports efficient energetic buffering during early adulthood. Future studies should also account for environmental variables such as seasonal conditions and barometric pressure, which have recently been shown to influence developmental timing and body mass [62].

Finally, the developmental trajectories described here provide an essential physiological baseline for interpreting responses to environmental stressors. Anthropogenic factors such as microplastics [44] and pesticides [43] are known to disrupt age- and sex-specific physiological processes, frequently reducing or eliminating natural sexual dimorphisms [63]. Establishing normative developmental profiles therefore represents an important prerequisite for identifying stress-induced deviations. Nevertheless, the physiological equilibrium observed at day 7 should be considered a transient developmental state rather than a permanent endpoint. Ageing in *T. molitor* is accompanied by progressive immunosenescence and reproductive decline [38,59], including a reduction in circulating haemocyte abundance and cellular function by approximately day 30 [53]. Extending longitudinal studies beyond the first week of adult life will therefore be essential to determine how long this mature physiological state is maintained and how energetic allocation, immune competence, and reproductive investment continue to interact throughout the adult lifespan.

## 5. Conclusions

This study demonstrates that the 7 days following adult eclosion represents a critical phase of integrated physiological remodelling in *T. molitor*, during which energetic reserves, cuticle maturation, and immune competence are coordinately reorganized to establish adult functional competence. Newly emerged beetles undergo an energetically demanding transition characterized by temporary body mass loss, rapid exoskeleton consolidation, and a marked expansion of circulating haemocytes, while maintaining stable humoral immune capacity. Although males and females ultimately achieve a comparable physiological state, our findings suggest that they reach this endpoint through distinct resource allocation strategies, with males exhibiting evidence of a trade-off between cuticle maturation and humoral immune potential. Together, these results demonstrate that adult eclosion marks the beginning, rather than the completion, of physiological maturation. By defining the developmental trajectories that characterize this post-eclosion window, our study provides a robust physiological baseline for *T. molitor*, with important implications for insect mass-rearing, experimental standardization, and future investigations into nutritional, environmental, and anthropogenic factors influencing adult performance. More broadly, our findings highlight early adulthood as a previously underappreciated developmental phase in which the coordination of energetic investment and immune maturation shapes the establishment of a fully functional adult phenotype.

## Data Availability

The original data presented in the study are openly available in the public Mendeley Data repository https://doi.org/10.17632/h8n5tyzpwk.1.
